# Accelerated preprocessing of large numbers of brain images by parallel computing on supercomputers

**DOI:** 10.1038/s41598-023-46073-4

**Published:** 2023-11-14

**Authors:** Takehiro Jimbo, Hidetoshi Matsuo, Yuya Imoto, Takumi Sodemura, Makoto Nishimori, Yoshinari Fukui, Takuya Hayashi, Tomoyuki Furuyashiki, Ryoichi Yokoyama

**Affiliations:** 1 Japan Research Activity Support Inc., Kobe, Japan; 2 Department of Urology, Kobe University Graduate School of Medicine, Kobe, Japan; 3 Laboratory for Brain Connectomics Imaging, RIKEN Center for Biosystems Dynamics Research, Kobe, Japan; 4 Department of Radiology, Kobe University Graduate School of Medicine, Kobe, Japan; 5 Mediest Co., Kobe, Japan; 6 Division of Molecular Epidemiology, Kobe University Graduate School of Medicine, Kobe, Japan; 7 Department of Mathematics, Faculty of Science, Tokyo University of Science, Tokyo, Japan; 8 Department of Brain Connectomics, Kyoto University Graduate School of Medicine, Kyoto, Japan; 9 Division of Pharmacology, Graduate School of Medicine, Kobe University, Kobe, Japan; 10 Department of Extended Intelligence for Medicine, The Ishii-Ishibashi Laboratory, Keio University, 35 Shinanomachi, Shinjuku-ku, Tokyo, 160-8582 Japan; 11 Yokoyama Lab, Tokyo, Japan

**Keywords:** Neuroscience, Computer science

## Abstract

“Preprocessing” is the first step required in brain image analysis that improves the overall quality and reliability of the results. However, it is computationally demanding and time-consuming, particularly to handle and parcellate complicatedly folded cortical ribbons of the human brain. In this study, we aimed to shorten the analysis time for data preprocessing of 1410 brain images simultaneously on one of the world's highest-performing supercomputers, “Fugaku.” The FreeSurfer was used as a benchmark preprocessing software for cortical surface reconstruction. All the brain images were processed simultaneously and successfully analyzed in a calculation time of 17.33 h. This result indicates that using a supercomputer for brain image preprocessing allows big data analysis to be completed shortly and flexibly, thus suggesting the possibility of supercomputers being used for expanding large data analysis and parameter optimization of preprocessing in the future.

## Introduction

The technical evolution of brain image analysis methods has led to the advancement and understanding of brain structure, function, and connectivity in health and disease conditions in humans^1,2^. In recent years, large population neuroscience data of brain images, such as the Human Connectome Project (HCP), UK Biobank (UKB), and Brain/MINDS-beyond, are being run and the data are made publicly available as open data^3–7^. Brain image analysis using deep learning and other data-reliant methods is expected to advance rapidly^8–11^, thus making it computationally demanding and requiring efficient analytical methods.

There are challenges in analyzing a large number of brain images. One of these is the computation time required for preprocessing, which includes standardized analysis for handling the variability of brain size and shape between individuals^12–14^. This preprocessing involves parcellation of the brain and cortical ribbon, estimation of cortical surfaces, and alignment of brain images, all of which are computationally expensive.

FreeSurfer^10^, one of the most popular software programs for preprocessing brain images, requires half to 1 day to process one brain structural image on a personal computer (PC) with commonly used specifications. Preprocessing brain image data from thousands of images may require an unrealistically long time (over several years), thus making it difficult to apply the results to higher-level analysis such as deep learning for automated diagnosis of brain diseases. In addition, the scale of brain imaging data is increasing year by year, and distributed processing by cloud-based supercomputing will be necessary in the near future to better understand the complexity of the brain^15^. Thus, it is important to enable the available analyzing tools to run on the supercomputing system.

Therefore, we investigated the capability of parallel processing of brain images by using the supercomputer “Fugaku,” one of the most powerful computers in the world as of 2022^16^. Fugaku has been used for several studies, such as molecular dynamics and simulations of brain circuits in the field of biology^17–19^. We considered that the Fugaku may enable us to simultaneously preprocess a large number of brain images.

**Figure 1:**
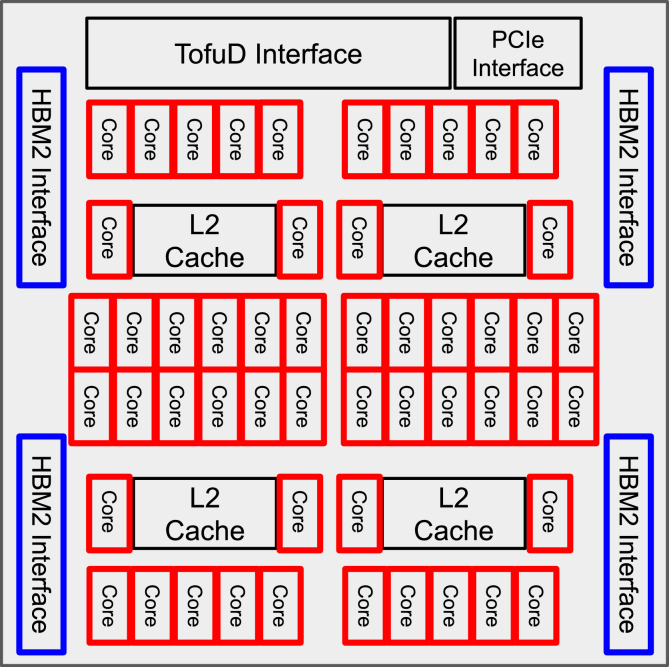
“Fugaku” node composition in a single node. Red boxes indicate cores, and blue boxes indicate memory. “Fugaku” has 48 threads and 32 GB memory per node. It has 158,976 nodes.

“Fugaku” has 158,976 nodes, and each node has 48 threads, 32 GB memory, and a 2 GHz clock frequency^20^ (Fig. 1). The key to parallelization and acceleration is the computational efficiency per node, which accelerates the overall computation by running multiple nodes. Therefore, in this study, we first focused on improving a single node’s performance of brain imaging preprocessing. Subsequently, by placing these processes on multiple nodes, a large amount of data processing is achieved in parallel. We also aimed to complete the preprocessing of over a thousand brain images in one day using “Fugaku.”

## Results

**Figure 2:**
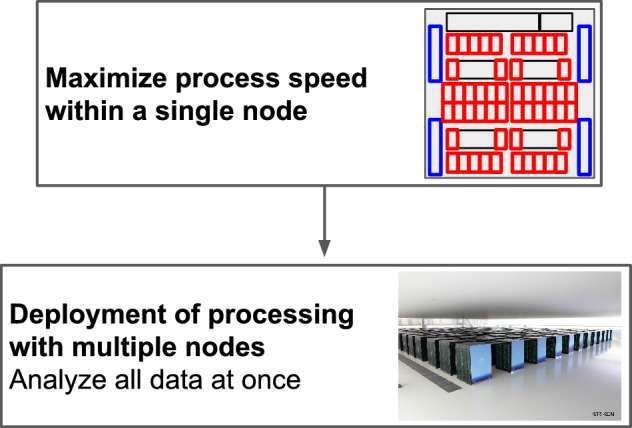
Summary of the strategy of speed-up methods. FreeSurfer processing was optimized and accelerated using a small amount of brain image data on a single node. Then, the process was extended to processing on multiple nodes to enable the processing of large numbers of brain images.

We used a two-step strategy to complete the preprocessing of over a thousand brain images in 1 day. First, FreeSurfer processing was optimized and accelerated using a small amount of brain image data from a single node. Second, the process was extended to processing multiple nodes to enable the processing of large numbers of brain images (Fig. 2). The results of these steps are presented below.

### First step: optimization and acceleration on a single node

We evaluated and optimized the parallelization of FreeSurfer processing on a single node. First, we evaluated and optimized thread parallelization in FreeSurfer processing (“each process in recon-all”) using a single brain image and then evaluated and optimized thread parallelization in multiple brain images.

#### Evaluation and optimization of thread parallelization in each “recon-all” process

**Figure 3:**
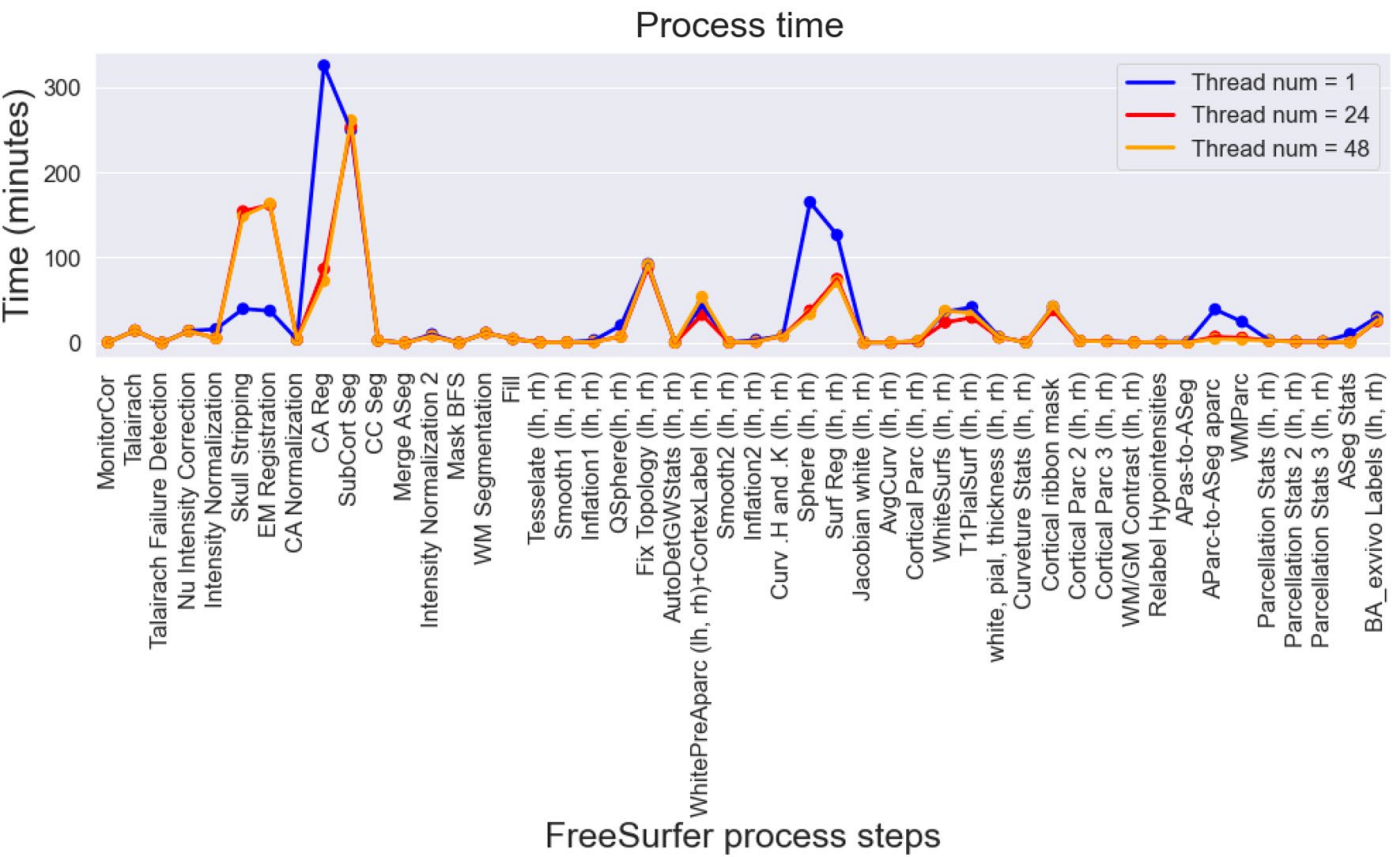
Plot of processing time for each FreeSurfer process at the single node to evaluate the number of threads using the process. The X-axis shows the recon-all process performed in FreeSurfer, and the Y-axis shows the elapsed time (min). Colors represent the number of threads used in the process. By parallelization, the “CA Reg,” “Sphere,” “Surf Reg,” “AParc-to-ASeg aparc,” and “WMParc” processes were confirmed to be faster. However, delays due to thread parallelization were observed in the “Skull Stripping” and “EM Registration” processes.

The processing speed of each processing step of “autorecon-all” was measured in FreeSurfer to evaluate thread parallelism on a single node; “autorecon-all” consists of 31 processes (Fig. 3). By parallelization, the “CA Reg,” “Sphere,” “Surf Reg,” “AParc-to-ASeg aparc,” and “WMParc” processes were confirmed to be faster. However, delays due to thread parallelization were observed in the “Skull Stripping” and “EM Registration” processes. We computed “Skull Stripping” and “EM Registration” in a single thread based on these results.

#### Evaluation and optimization of thread parallelization on multiple brain images

**Figure 4:**
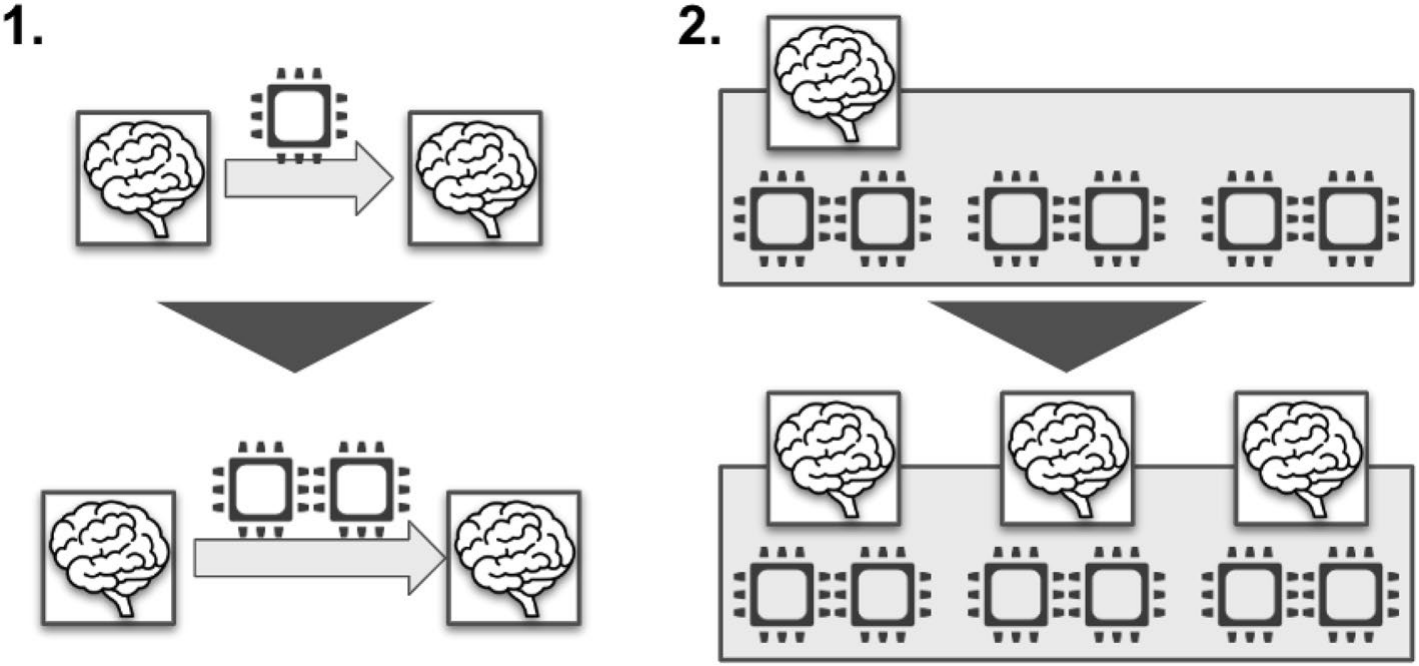
Consideration of the efficient calculations on a single node. In this study, we first focused on computation on a single node and examined how to efficiently perform computation on a single node. Specifically, as shown in the left figure, we examined the improvement in processing speed when the number of threads used for processing was increased for single-brain image data. Next, as shown in the figure on the right, we examined parallel processing, in which a single node processed multiple images simultaneously.

In this study, we aimed to process a large number of brain images simultaneously at high speed. The problem to be optimized was the number of threads that should be used to analyze the brain images of one person. The more threads used, the shorter the processing time, but the fewer the number of people who can be analyzed simultaneously. Therefore, we first varied the number of threads used for a single brain image from one thread (no thread parallelism) to 48 threads (all threads in one node) to measure the reduction in the processing time with the number of threads (Fig. 4).

**Figure 5:**
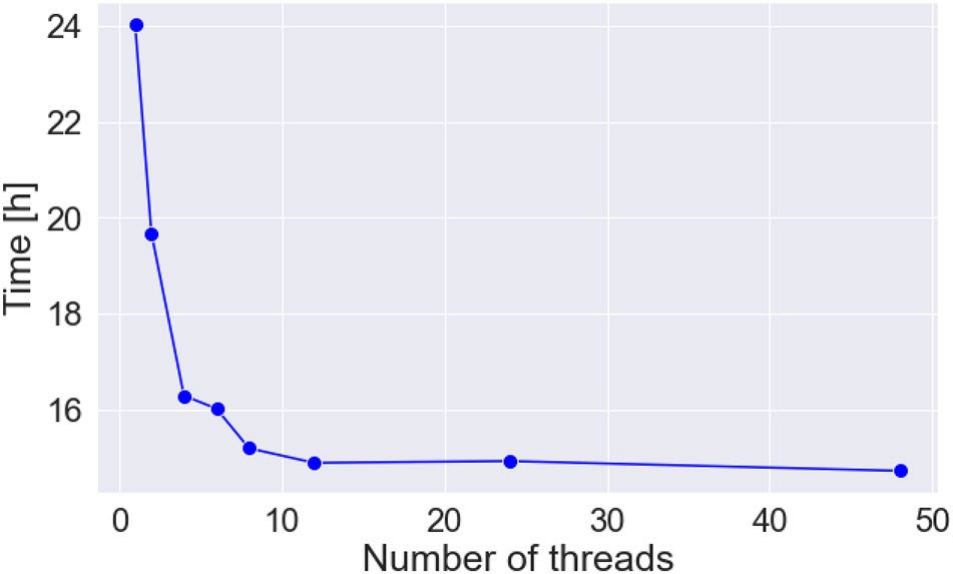
Effects of thread number on process time. The processing speed improved when the number of threads used for processing was increased for single brain image data on a single node. The horizontal axis shows the number of threads used per subject, and the vertical axis shows the processing time.

With a single thread, the process required 24 h; however, with eight threads in parallel, the time could be reduced to approximately 15 h. However, parallelization with more than eight threads did not improve the efficiency any further (Fig. 5). Therefore, we determined that thread parallelism is effective in reducing the processing time of one subject and that the use of less than eight threads is efficient (effective parallelization ratio, 51.33%).

**Figure 6:**
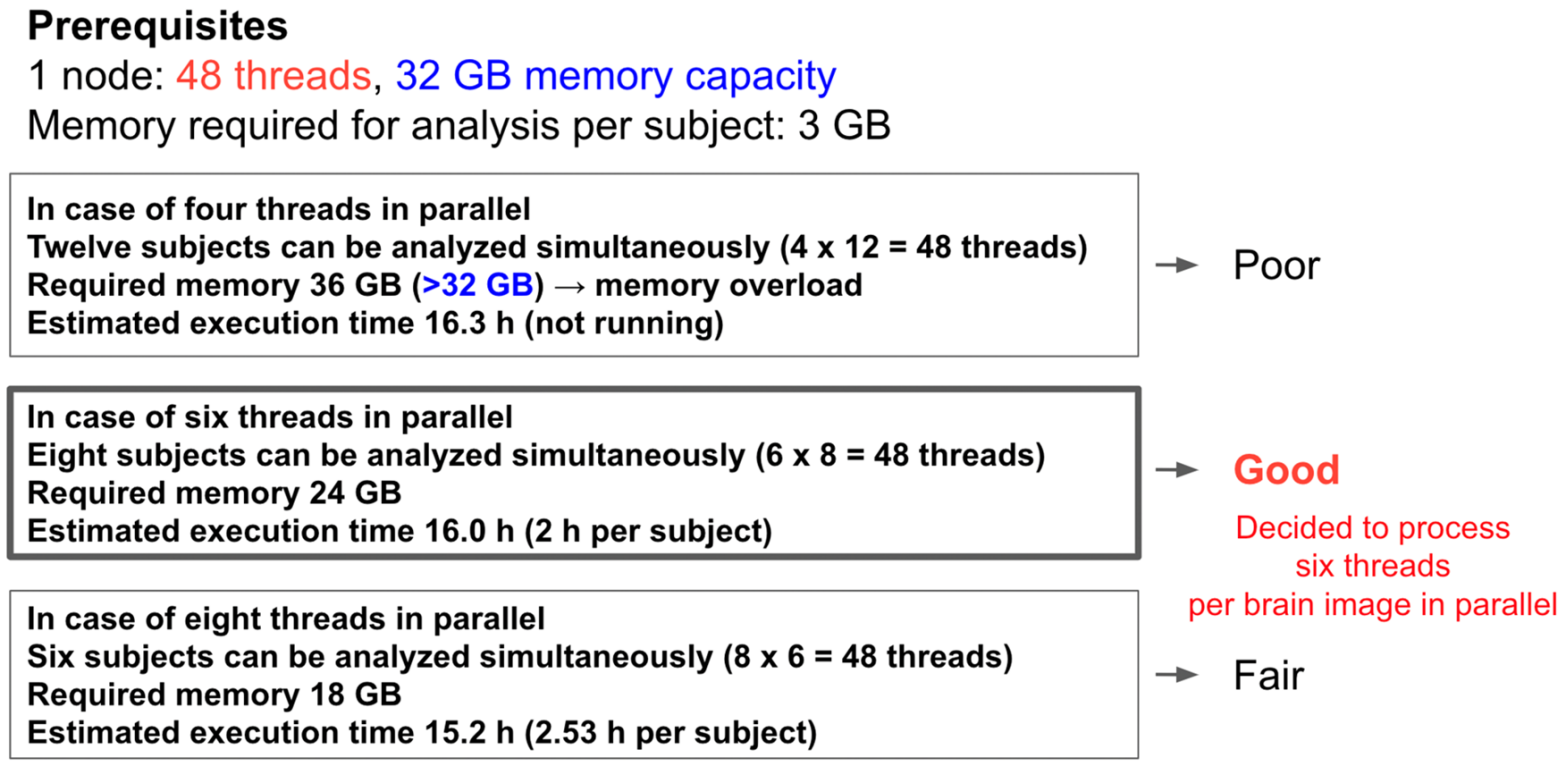
Comparison of computational optimization on a single node. There is a trade-off between the number of parallel threads used per brain image and the amount of data that can be processed simultaneously. It was found to be most efficient in using six threads per brain image (in this case, eight subjects' brain images could be processed simultaneously on a single node).

“Fugaku” has 48 threads per node and 32 GB of memory. The memory required for analysis per subject is approximately 3 GB. Based on this assumption, we compared the number of subjects that can be analyzed simultaneously, the required memory, and the estimated execution time. First, with four threads in parallel, 12 subjects can be analyzed simultaneously using all 48 threads on one node. The analysis of 12 subjects requires 36 GB of memory, which is not feasible because it will exceed the memory capacity per node. When comparing six-thread parallel and eight-thread parallel, eight-thread parallel has a shorter execution time of less than 1 h, but six-thread parallel can analyze two more subjects simultaneously. Moreover, the six-thread parallel is most efficient when viewed per subject. Therefore, we adopted the six-thread parallel and eight-subject-simultaneous analysis (Fig. 6).

### Second step: extension to multi-node processing

The efficient single-node processing (simultaneous processing of eight brain images per node) considered above was extended to multi-node. Computations were performed on multiple nodes for 1410 brain image data using 177 nodes. Consequently, the total computation time was completed in 17.22 h. The effective parallelization ratio and parallel efficiency were 99.98% and 95.84%, respectively. The 1410 brain image data in SRPBS were obtained with different scanners and acquisition protocols with different parameters. Differences in recon-all processing time for different protocols and the details of the recon-all results of multiple runs on the same subject are provided in the Supplementary Material.

## Discussion

In this study, we used the "Fugaku" supercomputer to accelerate brain image preprocessing using FreeSurfer for cortical reconstructions of 1410 magnetic resonance imaging (MRI) brain image data. To utilize “Fugaku's” large number of nodes and parallelization capability, we first focused on only one node to achieve speed-up and optimization within one node. Subsequently, by expanding the process to multiple nodes, we observed that the preprocessing of 1410 brain MRI data with FreeSurfer was completed in 17.33 h. Calculating brain structural imaging data of eight persons per node was the most efficient ratio for parallel computations with FreeSurfer.

The significance of this study is that it allows us to fully examine the analysis parameters of pretreatment using FreeSurfer and to respond flexibly to software updates. Until recently, it has been difficult to study and reanalyze preprocessing conditions when updating software because of the analysis time required by FreeSurfer. However, by applying the method used in this study, it is now possible to perform preprocessing with the latest software version, considering the most appropriate parameters for the data.

The use of supercomputers can facilitate research in smaller laboratories. In the research activities of a research institute, setting up a computer for each laboratory is costly and labor-intensive. Furthermore, preprocessing is time-consuming because installing high-performance computers on a laboratory-by-laboratory basis is difficult. This research approach is expected to solve these problems using supercomputers that are maintained at the national level. It's worth noting that, with the adoption of job scheduling systems or general parallel computing libraries such as OpenMPI, the analysis can also be conducted on other supercomputers, public clouds, or cluster PCs.

**Figure 7:**
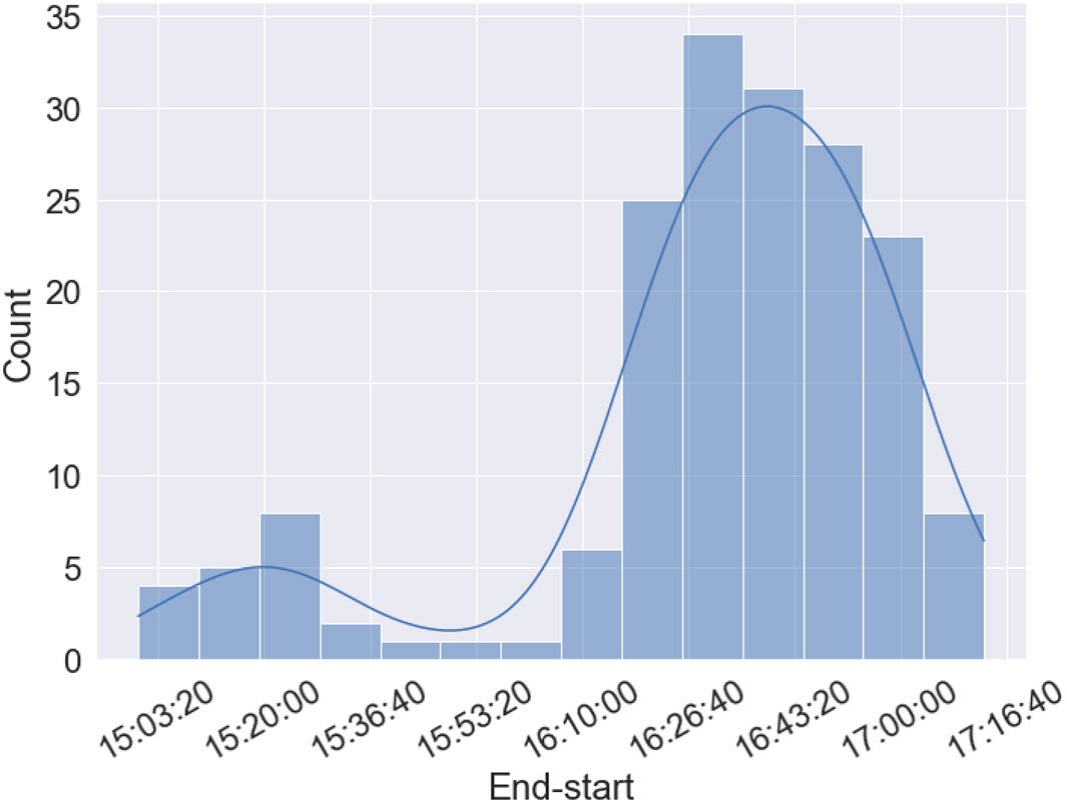
The completion time for each node in a multiple-node calculation. The difference in the start time from the end time is displayed on the x-axis. The completion time for all nodes, that is, the slowest node, was 17.33 h. Because all calculations on multiple nodes were performed on the same subject's brain images to remove the effects of data dependence from one subject to another, the fluctuations in the respective processing completion times are likely due to hardware factors in “Fugaku.”

Variations were observed in the execution time of each node of the multiple-node process, as shown in Fig. 7. In this study, processing was performed on one specific human brain image to avoid the influence of processing time owing to individual differences in images. Therefore, the variation in the processing time is most likely due to hardware problems. There are two possible reasons for this. First, the type of computation node used when allocating the processing to “Fugaku” may be a factor. “Fugaku” has two types of compute nodes: compute nodes, which are responsible only for job computation, and compute and Input/Output (IO) nodes, which are responsible for IO to the storage system in addition to computation. The compute node clocks at 2.0 GHz, and the compute and IO nodes execute the computation at a clock frequency of 2.2 GHz. Therefore, as the histogram in Fig. 7 shows, there are two peaks in the compute end time (one at approximately 16.5 h and the other approximately 10% shorter than the first one). The second factor is the possible file-copy time. This can also be explained by the system configuration of the compute node and compute and IO nodes. When the brain image data necessary for computation are copied from the storage system to the compute nodes, the data are first copied to the compute and IO nodes. Here, the files are copied to each compute node. Some computation nodes are directly connected to the compute and IO nodes, whereas others receive data from the compute and IO nodes via other compute nodes. Therefore, variations in computation time are expected to occur depending on the “distance” of the system configuration from the compute and IO nodes.

The effective parallelization ratio for 177 nodes was 99.98% in the current study. However, because the processing of the nodes is theoretically independent, it should be 100%. The reason the parallelization rate was below 100% in the demonstration experiment is believed to be because the calculation of the parallelization rate in this study involved a method that compares the average processing time of one node with the processing time that all 177 nodes completed (i.e., the processing time of the node that was the slowest). In this calculation, the parallelization rate was reduced by the processing time of the node, which was slower due to the variation described previously. However, because the variation in processing time per node that causes this decrease in parallelization rate is mainly due to the type of computation node, the decrease in parallelization rate is considered almost constant even if the number of parallelization is increased. Therefore, it is necessary to verify the parallelization rate in future studies using larger nodes.

Supplementary Table 2 in the Supplementary Material shows the between-subjects coefficient of variations (CVs) and the ratio of within-subjects to between-subjects CVs. It is important to note that the CV of one subject is close to zero. This means that the derived measurements are reliable even if FreeSurfer is run in parallel on a complex supercomputer.

In our study, we primarily focused on speeding up processing through parallelization of FreeSurfer. In contrast, Haddad et al. provided a detailed discussion on the reproducibility of statistical results, reporting differences in the results depending on the version of FreeSurfer used^21^. The environment used in this study is solely FreeSurfer version 7.2.0, and the platform is Red Hat Enterprise Linux 8.6. The analysis results of version 7.2.0 were not examined by Haddad et al.^21^; thus, we present new test–retest results.

This study had some limitations. First was the need to build software for ARM CPUs. Since most software programs are built for Intel, it is necessary to rebuild “Fugaku” from Intel to ARM. However, because “Fugaku” can use singularity containers^22^, we showed that the singularity environment can be easily reproduced if built on an ARM processor-based PC, such as the M1 Mac. Technical issues are also involved in the parallel execution of singularity, such as the creation of execution programs using the Message Passing Interface (MPI) and a parallel computation library. Second, among the FreeSurfer processes, Skull Stripping and EM Registration showed a significant decrease in computation speed when using Thread Parallel. Our analysis suggests that the file IO time constitutes a significant overhead, which could potentially impede system performance. Intriguingly, rather than enhancing performance as traditionally expected, the introduction of multiple parallel systems appears to inversely impact system efficiency. This deceleration may be attributable to the interplay between the FreeSurfer code and the file storage system. However, a comprehensive examination of such factors lies beyond the scope of the current study and presents substantial challenges for further validation due to their complex and interdependent nature. If the cause is clarified, further speeding up FreeSurfer is possible by parallel processing. Third, the effective parallelization ratio within a single node is 51.33%, which is not high. In other words, thread parallelism is effective for only approximately half of the processes. Therefore, regardless of the extent of the increase in the number of threads, one cannot expect a speed-up of more than a factor of 2. In this study, we only measured enabled thread parallelism and did not perform any tuning at the source level. It may also be possible to optimize the FreeSurfer build for “Fugaku” for faster processing. Therefore, performance improvement in the future may be possible if processes that are bottlenecks in processing time can be analyzed and areas where thread parallelism is effective can be determined.

In conclusion, we achieved parallelization and speed-up of preprocessing of multiple brain images using the supercomputer “Fugaku” in this study. Calculating brain structural imaging data of eight persons per node was the most efficient ratio for parallel computations with FreeSurfer for cortical surface reconstruction. Such parallel processing can be applied to distributed processing on cloud-based supercomputers in the future. This study will help in future brain imaging studies since the processing of large amounts of data is increasingly required in neuroscience.

## Methods

### Dataset

We used T1-weighted images from the SRPBS Multidisorder MRI Dataset as data^23^. The dataset consisted of 1410 T1-weighted images taken from multiple sites. In this study, data from one subject were randomly selected and used for analysis. The T1-weighted images of the subjects were copied and considered multiple subjects for analysis. In other words, the 1410 images used in the analysis were 1410 copies of brain images from one randomly selected subject. This was because we aimed to evaluate the effect of the supercomputer in brain image analysis and wanted to avoid confusion in evaluation indices, such as parallelization rate, due to differences in computation time caused by individual differences in brain data. In analysis, the images were transferred via the Internet in anonymized form. Anonymization was already performed in the open dataset using the ATR Deface Program^23^.

### FreeSurfer preprocessing

FreeSurfer, a standard software, was used as the preprocessing software for brain image analysis^23^. In neuroscientific research, the extensive utilization of FreeSurfer is notable. Nonetheless, due to its substantial processing duration, enhancing its computational efficiency is important. In this study, FreeSurfer version 7.2.0 was used. Singularity, a so-called Docker-like container platform for shared computers, such as supercomputers, was used to build the analysis environment. In addition, open-source software, such as FreeSurfer, is usually built to run on Intel CPUs, but the supercomputer “Fugaku” uses ARM CPUs. Therefore, in this study, the FreeSurfer source code was rebuilt for ARM.

In FreeSurfer analysis, Recon-all was mainly used in this study. Recon-all is a fully automated workflow that performs all of the FreeSurfer cortical reconstruction and sub-cortical segmentation steps in the following pipeline. First, Recon-all proceeds with corticometrics analysis that calculates the geometrical coordinates and surface thickness of the segmented brain for the brain surface topography. Then, Recon-all measures the volume, surface area, and average thickness of each brain region, the so-called intracranial volume analysis. Furthermore, Recon-all conducts histological classification of white matter, gray matter, and cerebrospinal fluid in the brain. This process extracts transformation matrices for each subject’s MRI. Ultimately, Recon-all generates high-density three-dimensional models for numerical brain simulations, where researchers can compare the mean value of geometrical quantities across groups (e.g., thickness, surface area, and volume).

### Computational resource

The supercomputer “Fugaku” was used in this study. “Fugaku” has 48 cores/48 threads per node, a 2.0 GHz clock frequency, and 32 GB memory. The total number of nodes in the “Fugaku” system is 158,976^20^. “Fugaku” has access restrictions for each user to ensure data security. It can be accessed if an application for use is submitted and accepted. For the execution of the job file “Fugaku”, a job file was created to analyze 8 subjects on one node and submitted 177 times.

### The parallel processing

Parallel processing was performed by developing a C++ wrapper program that executes singularities in parallel using the MPI.

### Calculation of parallelization rate by Gustafson’s law

The effective parallelization ratio was calculated to evaluate the parallel performance of parallel processing. The parallelization ratio is the ratio of the portion of a program that can be accelerated by parallel processing. In this experiment, Gustafson's law was solved using the measured values at two points with different numbers of parallelism to obtain the execution parallelization ratio. The parallelization ratio is denoted by α and is calculated as follows:

$$ \alpha = \frac{{nT_{m} - mT_{n} }}{{(1 - m)T_{n} - (1 - n)T_{m} }} $$

where n and m are the numbers of nodes at two points with different numbers of parallelisms (n ≥ 2 m), and Tn and Tm are the execution times at each number of nodes. In this study, m was 1; n, 177; T_m_, 59,329 s; and T_n_, 62,254 s.

### Supplementary Information

Supplementary Information.

## Data Availability

We used open-access data in this study. The data used are described in the “Methods” section. They are available from the SRPBS Multidisorder MRI Dataset and can be accessed by registering with the DecNef Brain Database Project at https://bicr-resource.atr.jp/srpbsopen/.
