# Author Correction: Accelerated preprocessing of large numbers of brain images by parallel computing on supercomputers

**DOI:** 10.1038/s41598-024-52774-1

**Published:** 2024-01-26

**Authors:** Takehiro Jimbo, Hidetoshi Matsuo, Yuya Imoto, Takumi Sodemura, Makoto Nishimori, Yoshinari Fukui, Takuya Hayashi, Tomoyuki Furuyashiki, Ryoichi Yokoyama

**Affiliations:** 1 Japan Research Activity Support Inc., Kobe, Japan; 2 Department of Urology, Kobe University Graduate School of Medicine, Kobe, Japan; 3 Laboratory for Brain Connectomics Imaging, RIKEN Center for Biosystems Dynamics Research, Kobe, Japan; 4 Department of Radiology, Kobe University Graduate School of Medicine, Kobe, Japan; 5 Mediest Co., Kobe, Japan; 6 Division of Molecular Epidemiology, Kobe University Graduate School of Medicine, Kobe, Japan; 7 Department of Mathematics, Faculty of Science, Tokyo University of Science, Tokyo, Japan; 8 Department of Brain Connectomics, Kyoto University Graduate School of Medicine, Kyoto, Japan; 9 Division of Pharmacology, Graduate School of Medicine, Kobe University, Kobe, Japan; 10 Department of Extended Intelligence for Medicine, The Ishii-Ishibashi Laboratory, Keio University, 35 Shinanomachi, Shinjuku-ku, Tokyo, 160-8582 Japan; 11 Yokoyama Lab, Tokyo, Japan

Correction to: *Scientific Reports* 10.1038/s41598-023-46073-4, published online 14 November 2023

The original version of this Article contained an error in the Acknowledgements section. The Grant Number was incorrect.

“This work used computational resources of the supercomputer “Fugaku” provided by the RIKEN Center for Computational Science through the HPCI System Research Project (Project ID: hp210282). The work was partly supported by the Research Organization for Information Science and Technology (RIST) under the HPCI User Optimization Support Program and by grants from the Japan Agency of Medical Research and Development (AMED) (JP22gm0910012, JP22wm0425001) (T.F), (JP18dm0307006, JP19wm0525006) (T.H), Grants-in-Aids for Scientific Research from the Japan Society for the Promotion of Science (21H04812) (T.F), Grants-in-Aids for Scientific Research from the Ministry of Education, Culture, Sports, Science and Technology in Japan (18H05429) (T.F). This research was funded by the Hyogo COE Research Program and The New Industry Research Organization (NIRO). We would like to thank Dr. Koji Hanihara, Ms. Kaho Akiyoshi, and Mr. Toshikaze Chiba for their assistance in the analysis.”

now reads:

“This work used computational resources of the supercomputer “Fugaku” provided by the RIKEN Center for Computational Science through the HPCI System Research Project (Project ID: hp210282). The work was partly supported by the Research Organization for Information Science and Technology (RIST) under the HPCI User Optimization Support Program and by grants from the Japan Agency of Medical Research and Development (AMED) (JP22gm0910012, JP22wm0425001) (T.F), (JP18dm0307006, JP19dm0307004) (T.H), Grants-in-Aids for Scientific Research from the Japan Society for the Promotion of Science (21H04812) (T.F), Grants-in-Aids for Scientific Research from the Ministry of Education, Culture, Sports, Science and Technology in Japan (18H05429) (T.F). This research was funded by the Hyogo COE Research Program and The New Industry Research Organization (NIRO). We would like to thank Dr. Koji Hanihara, Ms. Kaho Akiyoshi, and Mr. Toshikaze Chiba for their assistance in the analysis.”

The original Article has been corrected.

