# Supplementary material for: Accelerated preprocessing of large numbers of brain images by parallel computing on supercomputers
Source: Sci Rep. 2023 Nov 14;13:19901. doi: 10.1038/s41598-023-46073-4 (PMC10646110; doi:10.1038/s41598-023-46073-4)
Supplement: Supplementary file 1 — Supplementary Information. [file 41598_2023_46073_MOESM1_ESM.docx]

**Supplementary Material**

**Differences in recon-all processing time for different protocols**

We conducted an analysis of the data obtained through various scanners and data acquisition protocols provided by the SRPBS Multidisorder MRI Dataset. The field strength for all protocols was 3T. Further details regarding the different scanner settings for each protocol are provided in the supporting materials.

The distribution of processing time for each protocol is represented in Supplementary Fig. 1. Upon examining the median values, it is apparent that Protocols 2, 3, and 7 require more time than the others. This could be because these protocols employ earlier versions of 3T MRI machines, which may result in comparatively lower image quality even at the same resolution. However, multiple factors could contribute to variations in quality between different MRI machines. The examination of these factors is beyond the scope of this study.

**Differences in output measures for 1410 recon-all runs on the same subject**

In this study, we performed 1410 recon-all processes on the same subject. The coefficient of variation (CV) for the segmentation volumes of subcortical structures obtained from the results of recon-all was calculated and compared for different subjects and multiple parallel runs on an individual (Supplementary Table 2). As a result, the calculations obtained from these runs on an individual were found to be very small (<0.6%) compared to those of different subjects. This indicates that the test-retest accuracy of the calculations in parallel runs is high.

| **Protocol** | **Site** | **Number of subjects** | **Scanner** | **Magnitic field strength** | **Voxel size**  **(mm^3)** | **TR (ms)** | **TE (ms)** | **TI (ms)** | **Flip angle (deg)** | **FOV** | **Matrix** |
| --- | --- | --- | --- | --- | --- | --- | --- | --- | --- | --- | --- |
| 1 | Showa University | 235 | SIEMENS Verio | 3T | 1 x 1 x 1 | 2300 | 2.98 | 900 | 9 | 256 | 256 x 256 |
| 2 | Hiroshima University  Hospital | 124 | GE Signa HDxt | 3T | 1 x 1 x 1 | 6812 | 1896 | 450 | 20 | 256 | 256 x 256 |
| 3 | Hiroshima University  Hospital | 65 | GE Signa HDxt | 3T | 1 x 1 x 1 | 6812 | 1896 | 450 | 20 | 256 | 256 x 256 |
| 4 | Hiroshima Kajikawa Hospital | 62 | SIEMENS Symphon | 3T | 1 x 1 x 1 | 1900 | 2.38 | 900 | 10 | 256 | 256 x 256 |
| 5 | Center of Innovation in  Hiroshima University | 195 | SIEMENS MAGNETOM Verio.Dot | 3T | 1 x 1 x 1 | 2300 | 2.98 | 900 | 9 | 256 | 256 x 256 |
| 6 | Kyoto university | 220 | SIEMENS TimTrio | 3T | 0.9375 x 0.9375 x 1.0 | 2000 | 3.4 | 990 | 8 | 225 x 240 | 240 x 256 |
| 7 | Kyoto university | 122 | SIEMENS Trio | 3T | 0.9375 x 0.9375 x 1.0 | 2000 | 4.38 | 990 | 8 | 225 x 240 | 240 x 256 |
| 8 | University of Tokyo | 272 | GE Discovery MR750w | 3T | 1 x 1 x 1.2 | 7.7 | 3.1 | 400 | 11 | 240 | 256 x 256 |
| 14 | CiNet | 63 | SIEMENS TimTrio | 3T | 1 x 1 x 1 | 1900 | 2.48 | 900 | 9 | 256 | 256 x 256 |

Supplementary Table 1 Details of each protocol

This table describes the settings of the different imaging protocols for the SRPBS Multidisorder MRI Dataset [23]. Protocols 9 to 13 have been excluded due to the small number of subjects involved. This table cites numerical values from the original paper. If you have any questions, please contact the authors of the original paper.

| **subcortical structures** | **CV (different subjects)** | **CV_0_ (same subject)** | **Ratio (CV_0_/CV)** |
| --- | --- | --- | --- |
| Lateral-Ventricle | 0.50673 | 0.00009 | 0.00018 |
| Inf-Lat-Vent | 0.60983 | 0.00037 | 0.00060 |
| Cerebellum-White-Matter | 0.14268 | 0.00003 | 0.00019 |
| Cerebellum-Cortex | 0.10692 | 0.00011 | 0.00107 |
| Thalamus | 0.12809 | 0.00005 | 0.00037 |
| Caudate | 0.21113 | 0.00002 | 0.00008 |
| Putamen | 0.17732 | 0.00008 | 0.00044 |
| Pallidum | 0.14404 | 0.00012 | 0.00081 |
| 3rd-Ventricle | 0.33151 | 0.00015 | 0.00046 |
| 4th-Ventricle | 0.27996 | 0.00000 | 0.00000 |
| Brain-Stem | 0.10545 | 0.00004 | 0.00036 |
| Hippocampus | 0.11317 | 0.00014 | 0.00125 |
| Amygdala | 0.13503 | 0.00081 | 0.00602 |
| CSF | 0.23936 | 0.00034 | 0.00143 |
| Accumbens-area | 0.26122 | 0.00096 | 0.00367 |
| VentralDC | 0.11085 | 0.00007 | 0.00066 |
| vessel | 0.58022 | 0.00028 | 0.00048 |
| choroid-plexus | 0.30918 | 0.00000 | 0.00000 |
| WM-hypointensities | 2.33370 | 0.00933 | 0.00400 |
| Optic-Chiasm | 0.35169 | 0.00029 | 0.00083 |
| CC_Posterior | 0.17846 | 0.00057 | 0.00321 |
| CC_Mid_Posterior | 0.19521 | 0.00046 | 0.00234 |
| CC_Central | 0.24717 | 0.00030 | 0.00121 |
| CC_Mid_Anterior | 0.28364 | 0.00049 | 0.00174 |
| CC_Anterior | 0.19488 | 0.00081 | 0.00417 |

Supplementary Table 2 Coefficient of variations of subcortical segmentation volumes

This table describes the coefficient of variation (CV) for the segmentation volumes of subcortical structures obtained from FreeSurfer's recon-all process results. The CV for results across different subjects and the CV for multiple runs on the same subject (CV_0_) were calculated; the Ratio above represents the comparison of the two CVs (CV_0_/CV).


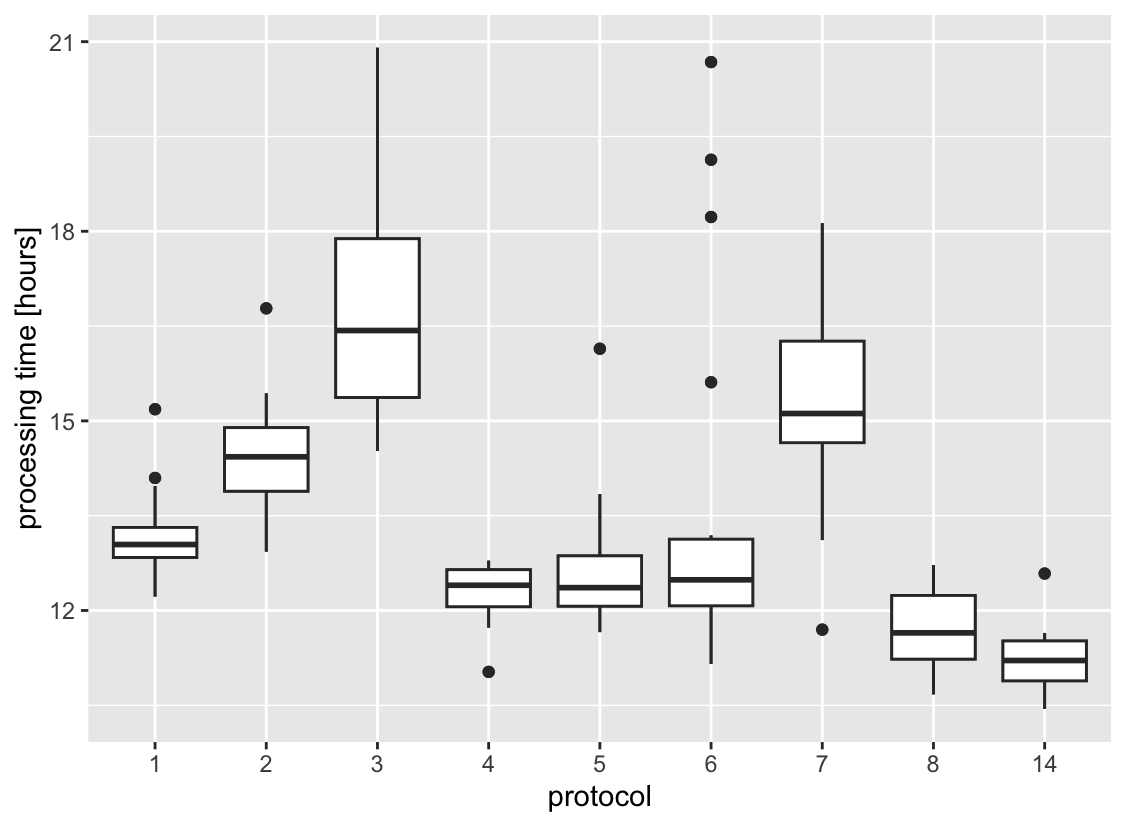


Supplementary Fig. 1 Boxplot of Computation Time by Protocol

The processing time of recon-all for each protocol of SRPBS is represented in a boxplot. Details of each protocol are described in Supplementary Table 1. Protocols with fewer than 40 subjects were excluded.
